# Salvage esophagectomy for local recurrent esophageal cancer after definitive chemoradiotherapy followed by photodynamic therapy: A case report

**DOI:** 10.1016/j.ijscr.2021.02.003

**Published:** 2021-02-03

**Authors:** Wataru Hirose, Yusuke Taniyama, Fumiyoshi Fujishima, Chiaki Sato, Michiaki Unno, Takashi Kamei

**Affiliations:** a Department of Surgery, Tohoku University Graduate School of Medicine, 1-1 Seiryo-machi, Aoba-ku, Sendai, Miyagi, 980-8574, Japan; b Department of Pathology, Graduate School of Medicine, Tohoku University, 1-1 Seiryo-machi, Aoba-ku, Sendai, Miyagi, 980-8574, Japan

**Keywords:** CRT, chemoradiotherapy, CT, computed tomography, FDG, 18F-fluorodeoxyglucose, PDT, photodynamic therapy, SqCC, squamous cell carcinoma, Oesophageal cancer, Photodynamic therapy, Salvage surgery

## Abstract

**Introduction:**

Photodynamic therapy (PDT) is performed as a salvage treatment for patients with residual or recurrent esophageal cancer after chemoradiotherapy (CRT). Although PDT is considered less invasive than salvage surgery, it is unclear how deep its effects are and whether it causes damage to adjacent tissues. Herein, we report a case of esophageal cancer treated with PDT followed by esophagectomy. In this case, we evaluated the effect of PDT on adjacent tissues based on surgical and pathological examination.

**Presentation of case:**

A 58-year-old man with dysphagia was diagnosed with esophageal squamous cell carcinoma (SqCC; T1N0M0, Stage I) in the upper thoracic esophagus. He underwent definitive CRT with two courses of 5-fluorouracil and cisplatin every 4 weeks with 60 Gy of radiation. Twelve months after CRT, endoscopic examination revealed local recurrence, and PDT using talaporfin sodium was performed. The tumor recurred again 6 months after PDT, and robot-assisted thoracoscopic esophagectomy was performed as a definitive treatment. Tissues around the left side of the esophagus and thoracic duct were tightly adherent with severe fibrosis and were successfully removed by extended resection. Histopathological examinations showed that the esophageal wall and peri-esophageal tissue were replaced by fibrous tissue and this extended even beyond the tumor.

**Discussion:**

The primary tumor was limited to the submucosal layer, and the target for irradiation had some longitudinal margins. Therefore, PDT can cause intense inflammation in tissues adjacent to the tumor.

**Conclusions:**

It is necessary to consider the location when performing salvage esophagectomy after PDT.

## Highlights

• We report a case of esophageal cancer treated with PDT followed by esophagectomy.
• We assessed the PDT effect on adjacent tissues based on surgery and pathology.
• PDT can cause intense inflammation in tissues adjacent to the tumor.
• The location should be considered when performing salvage esophagectomy after PDT.

## 1 Introduction

Esophageal cancer is one of the most aggressive gastrointestinal malignancies. It is the sixth leading cause of cancer-related death in men [1]. Photodynamic therapy (PDT) is a salvage treatment used in patients with local recurrent esophageal cancer after chemoradiotherapy (CRT) [[2], [3], [4], [5]]. This treatment offers the only chance of achieving complete cancer remission for patients who are not candidates for salvage surgery. The overall local complete response rate of PDT was 62.1 % among cases of recurrent esophageal cancer after CRT [6]. Although PDT is less invasive than salvage surgery, it could lead to fatal adverse events such as aortic-esophageal fistula [4]. The treatment effect of PDT on the tumor appears to extend to the proper muscle layer at most [7,8]. However, it is still unclear how PDT affects adjacent normal tissues deeper than the proper muscle layer. To date, few patients have undergone esophagectomy for recurrent esophageal cancer after PDT [4]. Here, we report a case of salvage esophagectomy after PDT and evaluate the effect of PDT on the esophagus and its surrounding tissues using surgical and histopathological examinations. This article has been reported in line with the SCARE criteria [9].

## 2 Presentation of case

**Fig. 1:**
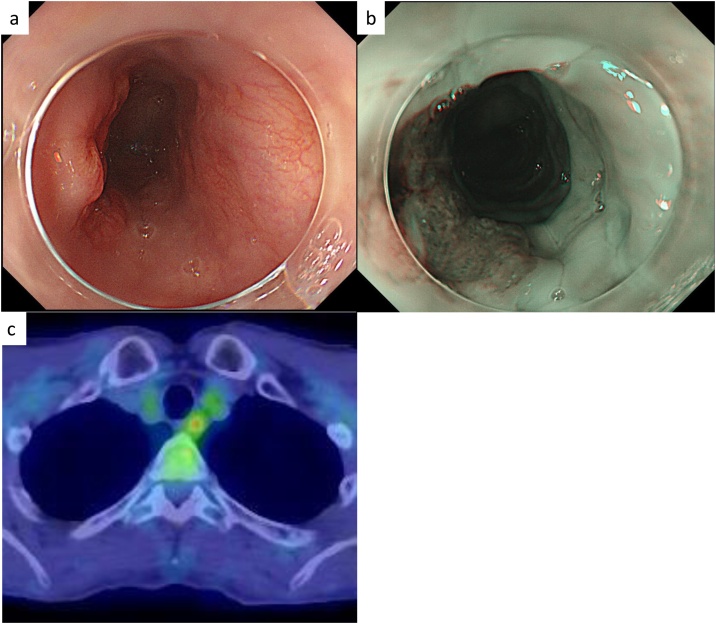
Examination before chemoradiotherapy. a: Upper gastrointestinal endoscopy showing a type 0-IIa + IIc elevated lesion located 22–23 cm from the incisors. b: Narrow-band imaging showing a brownish area at the elevated lesion. c: 18F-deoxyglucose (FDG) positron emission tomography showing high FDG uptake in the thickened wall.

We describe the case of a 58-year-old man who underwent upper gastrointestinal endoscopy for dysphagia in 2017. The examination showed a type 0-IIa + IIc elevated lesion located 22–23 cm from the patient’s incisors (Fig. 1a), and narrow-band imaging showed a brownish area (Fig. 1b). Pathological examination of the tumor biopsy showed squamous cell carcinoma (SqCC). Enhanced computed tomography (CT) could not detect the primary lesion and lymph node or distant metastases. 18F-ﬂuorodeoxyglucose (FDG) positron emission tomography showed relatively high FDG uptake (SUV max 5.0) in the upper esophagus (Fig. 1c). The patient was diagnosed with esophageal SqCC in the upper thoracic esophagus, classified as clinical Stage I (cT1N0M0) according to the UICC-TNM classification 8th edition [10]. Since the patient was not eligible for endoscopic resection, he underwent definitive CRT with two courses of 5-fluorouracil (700 mg/m^2^) and cisplatin (70 mg/m^2^) every 4 weeks with 60 Gy of radiation.

**Fig. 2:**
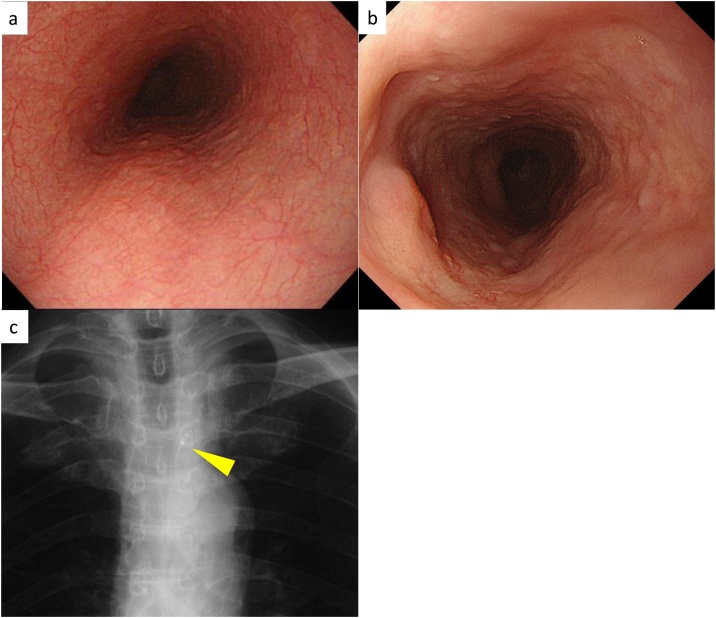
Upper gastrointestinal endoscopy after chemoradiotherapy. a: Examination 1 month after chemoradiotherapy. The patient achieved complete remission. b: Upper gastrointestinal endoscopy before photodynamic therapy showing an elevated lesion at the previous treatment area 12 months after complete remission. C: Chest radiograph before photodynamic therapy. A marker clip (arrowheads) was placed on the oral side of the tumor at the clavicular notch.

**Fig. 3:**
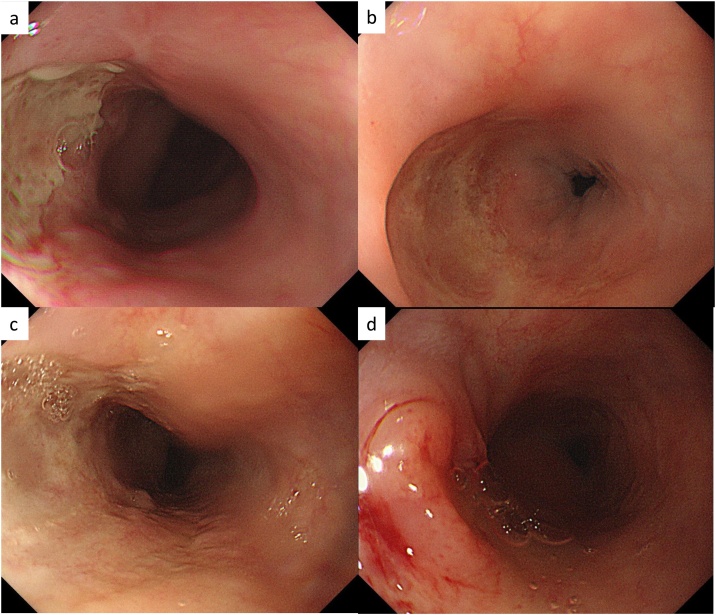
Upper gastrointestinal endoscopy after photodynamic therapy. a: An ulcerated scar with white moss attached 3 weeks after photodynamic therapy. b: An ulcerated scar 5 weeks after photodynamic therapy. c: An ulcerated scar 7 weeks after photodynamic therapy. d: A recurrence of the tumor was detected 5 months after photodynamic therapy.

The patient achieved complete remission of the lesion for 12 months (Fig. 2a). However, endoscopic examination revealed an elevated lesion at the previous treatment area (Fig. 2b) and a biopsy showed SqCC. We diagnosed the patient with local recurrence after CRT. The tumor was located in the upper thoracic esophagus (Fig. 2c). Given that we were unable to detect the tumor on CT, and accumulation on positron emission tomography was weak, the depth of the tumor invasion was diagnosed as T1b [10] based on the endoscopic findings. There was no metastasis to any other lymph node or organ. Because the patient was hesitant to undergo salvage esophagectomy, PDT was performed for salvage treatment. The laser was irradiated through an endoscopic device 4 h after the intravenous injection of talaporfin sodium (65 mg/body, 40 mg/m^2^). The amount of irradiated laser was 150 J/cm^2^ × 2. The patient had no side effects, including hoarseness, after PDT. The tumor disappeared macroscopically (Fig. 3a–c), and a biopsy did not show malignant cells. However, once again, local recurrence was detected 6 months after PDT (Fig. 3d), and classified as clinical Stage II (cT2N0M0) [10]. The patient finally consented to undergo surgical resection.

**Fig. 4:**
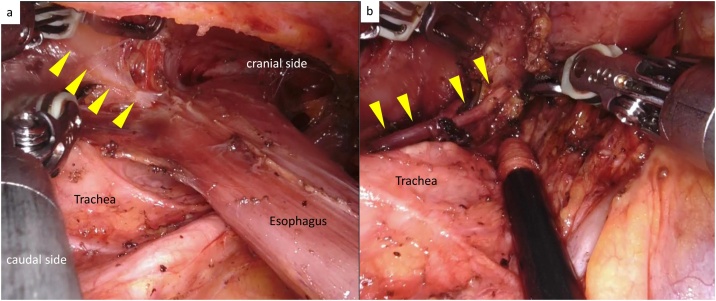
Operation findings on the left side of the esophagus. a: The thoracic duct (arrowheads) was fibrosed and had to be resected. b: The left recurrent laryngeal nerve (arrowheads) tightly adheres to the esophagus.

Robot-assisted thoracoscopic esophagectomy was performed in the semi-prone position. Although the right side of the esophagus was slightly edematous, fibrotic change was not observed in the upper esophagus. However, on the left side of the upper esophagus, tissues around the esophagus and thoracic duct were tightly adherent with intent fibrosis (Fig. 4a). The thoracic duct was clipped at the caudal aspect of the fibrous tissue and removed with the esophagus and its surrounding tissue. The left recurrent laryngeal nerve was also tightly adherent to the esophagus (Fig. 4b) but was preserved by sharp dissection. After the thoracoscopic procedure, the patient was placed in the supine position. A gastric conduit was created and raised through the posterior mediastinal route using hand-assisted laparoscopic surgery. Anastomosis of the gastric conduit to the cervical esophagus was performed by a hand-sewn procedure on the left side of the neck. Although anastomotic leakage was detected on CT 10 days after surgery, the patient was treated conservatively and discharged 46 days after surgery.

**Fig. 5:**
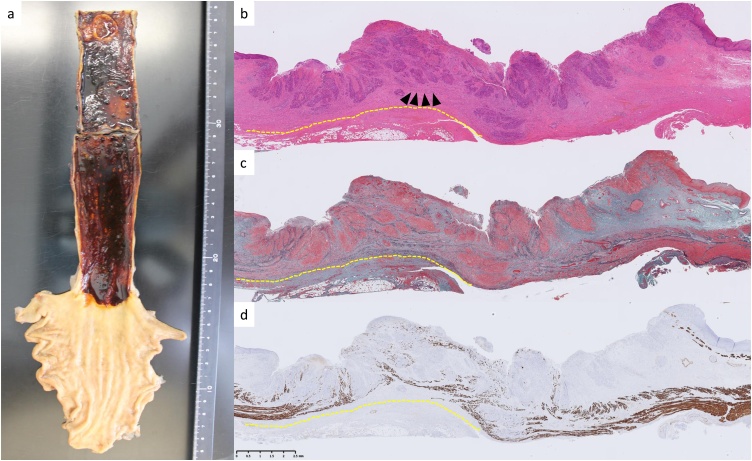
Macroscopic and histopathological findings. a: Macroscopic findings. The tumor is located in the upper esophagus and is a type 2 lesion. b: Hematoxylin and eosin-stained section. The arrowheads indicate the deepest part of the tumor. The dotted line is the adventitia of the esophagus. c: Elastica–Masson staining showing fibrotic change in the esophageal wall and adipose tissue around the tumor. Fibrous change is not detected away from the tumor. d: Immunohistological staining for desmin showing the muscle layer. The external longitudinal muscles are barely visible.

Macroscopically, the tumor was located in the upper esophagus and was a type 2 lesion (Fig. 5a). Microscopically, the structure of the esophageal wall was difficult to detect because of the tumor proliferation and fibrosis. In addition, the depth of the invasion was confirmed by performing immunohistochemical staining for desmin. Although the cancer cells were observed at a level slightly deeper than the proper muscle layer, the surgical margin was negative. Lymph-node metastasis was not detected. The tumor was defined as pathological Stage II (pT3N0M0) according to the TNM criteria. A significant pathological finding in this case is that the peri-esophageal adipose tissue away from the tumor involved fibrosis (Fig. 5b). This finding became more apparent with Elastica–Masson staining (Fig. 5c) and immunohistological staining for desmin (Fig. 5d) to detect the original layer of proper muscle. After salvage surgery, the patient had no recurrence for 5 months.

## 3 Discussion

According to the Guidelines for Diagnosis and Treatment of Carcinoma of the Esophagus 2017 published by The Japan Esophageal Society, PDT is considered as a salvage treatment option for patients with residual or recurrent cancer after CRT [2]. PDT is effective for tumors with a depth limited to the muscle layer [11]. Moreover, PDT is indicated for patients who are not eligible for or willing to undergo salvage surgery [4,5]. Therefore, surgical resection is not usually performed after PDT.

The level of inflammation and/or fibrous change in surrounding tissues caused by PDT is unclear. There are a few reports on histopathological changes after PDT. Although Yano et al. reported salvage surgeries for five patients after PDT [4], surgical or histopathological findings were not included in their report. Horimatsu et al. reported that tissue injury due to PDT extends to the muscle layer or even deeper, as demonstrated in a canine model [8]. In their report, the spread of inflammation caused by PDT to peri-esophageal tissues was observed during surgery, and they proposed that tissue changes were not only due to PDT but also due to tumor necrosis based on the histopathological findings.

Another report that documented aortic-esophageal fistula following PDT indicated that this phenomenon was induced by radiation therapy to the tumor [4]. In our case, however, the primary tumor was limited to the submucosal layer. Therefore, tumor necrosis should not have occurred in the muscle layer or deeper. In addition, because the target tumor volume for irradiation should have had some longitudinal margins [12], it was unlikely that fibrous change would be limited to the tumor site if it was caused by radiation. We propose that fibrous change of the peri-esophageal tissue can be explained by the effect of PDT, not by that of irradiation.

Another issue to discuss is whether this fibrotic change might have been caused by the cancer cells. The pathological findings in this case revealed that the typical fibrotic change occurred around the tumor, not within the tumor. The cancer that causes fibrotic changes usually generates fibrous tissue inside of the tumor. In addition, the morphological pattern of invasion in those cases are infiltrative and have unclear borders. Thus, our pathologist theorized that these fibrotic changes were not caused by the cancer cells.

In terms of operative findings, the tissue around the esophagus and thoracic duct on the left side of the esophagus was severely fibrosed. The thoracic duct showed fibrous tissue involvement and had to be resected. However, if this adjacent tissue had been an organ, such as the aorta or trachea, surgical resection would have been impossible and would not have been considered. For this reason, when performing PDT, it is necessary to pay attention to the anatomical relationship to the surrounding organs, regardless of the depth of invasion.

In the future, the number of patients who undergo esophagectomy after PDT may increase as PDT becomes more common. We would like to emphasize that while PDT is a non-surgical modality and a generally safe procedure, it is important to envision the organ beyond the esophageal wall where there is the potential for irradiation from the laser. Severe inflammation and adhesion between this adjacent organ and the esophagus would render esophagectomy exceptionally difficult.

## 4 Conclusion

PDT can cause intense inflammation beyond the esophageal tissue around the tumor regardless of the depth of the tumor. It is necessary to consider the anatomical relationship with the surrounding tissues when performing salvage esophagectomy after PDT.

## Declaration of Competing Interest

The authors report no declarations of interest.

## Source of funding

This research did not receive any specific grant from funding agencies in the public, commercial, or not-for-profit sectors.

## Ethics approval

Ethical approval has been exempted from our institution for this case report.

## Consent

Written informed consent was obtained from the patient for publication of this case report and accompanying images. A copy of the written consent is available for review by the Editor-in-Chief of this journal on request.

## Authors’ contributions

WH contributed to the writing of the manuscript. YT participated in its design and coordination and helped to draft the manuscript. FF supervised the work as pathologists. YT and CS performed the surgery. MU and TK supervised the manuscript. All authors read and approved the final manuscript.

## Registration of research studies

Not applicable.

## Guarantor

Takashi Kamei accepted full responsibility for the work and had controlled the decision to publish.

## Provenance and peer review

Not commissioned, externally peer-reviewed.
